# Supplementary material for: Identification of photoperiod-induced specific miRNAs in the adrenal glands of Sunite sheep (Ovis aries)
Source: Front Vet Sci. 2022 Jul 22;9:888207. doi: 10.3389/fvets.2022.888207 (PMC9354845; doi:10.3389/fvets.2022.888207)
Supplement: Supplementary file 1 [file Data_Sheet_1.ZIP › Supplementary file/miRDeep2 file.zip!/miRDeep2 file/Know_Structure/LP42A2/oar-mir-1193.pdf]

miRBase precursor	:	oar-mir-1193
Total read count	:	5
oar-miR-1193-5p read count	:	4
oar-miR-1193-3p read count	:	0
remaining reads	:	1

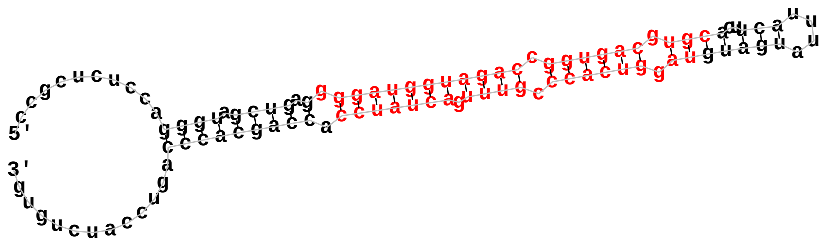

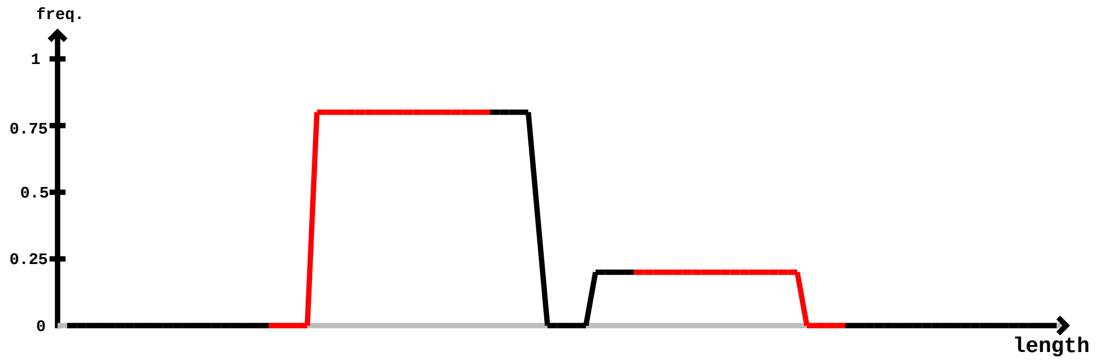


```
                              oar-miR-1193-5p
                                                                       oar-miR-1193-3p
5'- ccgcucuccaggguagcugaggggaugguagaccggugacgugcacuucauuuaugauguaggucacccguuugacuauccaccagcacccaguccaucugug   -3'    exp
    ..........((((.((((.(.(((((((((((.((((((.((((..(((....))))))).)))))).)))).))))))).))))))))).........    reads      mm          sample
    .........................ugguagaccggugacgugcacuu........................................................   2          0           seq
    .........................ugguagaccggugacgugcacuuc.......................................................   2          0           seq
    ......................................................ugauguaggucacccguuugac...........................   1          0           seq
```
